# Comparative Study of rK39 Leishmania Antigen for Serodiagnosis of Visceral Leishmaniasis: Systematic Review with Meta-Analysis

**DOI:** 10.1371/journal.pntd.0001484

**Published:** 2012-01-31

**Authors:** Zuinara Maia, Monique Lírio, Sóstenes Mistro, Carlos Maurício Cardeal Mendes, Sanjay R. Mehta, Roberto Badaro

**Affiliations:** 1 Department of Medicine, Federal University of Bahia Salvador, Bahia, Brazil; 2 Department of Medicine, University of California San Diego, La Jolla, California, United States of America; Emory University, United States of America

## Abstract

**Background:**

The rK39 recombinant protein is derived from a specific antigen produced by the *Leishmania donovani* complex, and has been used in the last two decades for the serodiagnosis of visceral leishmaniasis. We present here a systematic review and meta-analysis of studies evaluating serologic assays to diagnose visceral leishmaniasis to determine the accuracy of rK39 antigen in comparison to the use of other antigen preparations.

**Methodology/Principal Findings:**

A systematic review with meta-analysis of the literature was performed to compare the rK39 strip-test and ELISA formats against serological tests using promastigote antigens derived from whole or soluble parasites for Direct Aglutination Test (DAT), Indirect Immunofluorescence test (IFAT) and ELISA with a promastigote antigen preparation (p-ELISA). Gold standard diagnosis was defined by the demonstration of amastigotes on hematological specimens. A database search was performed on *Medline*, *Lilacs*, *Scopus*, *Isi Web of Science*, and *Cochrane Library*. Quality of data was assessed using the QUADAS questionnaire. A search of the electronic databases found 352 papers of which only 14 fulfilled the selection criteria. Three evaluated the rK39 ELISA, while 13 evaluated the rK39 immunochromatographic strip test. The summarized sensitivity for the rK39-ELISA was 92% followed by IFAT 88% and p-ELISA 87%. The summarized specificity for the three diagnostic tests was 81%, 90%, and 77%. Studies comparing the rK39 strip test with DAT found a similar sensitivity of 94%, although the DAT had a slightly higher specificity. The rK39 strip test was more sensitive and specific than the IFAT and p-ELISA. We did not detect any difference in the sensitivity and specificity between strips produced by different manufacturers.

**Conclusions:**

The rK39 protein used either in a strip test or in an ELISA, and the DAT are the best choices for implementation of rapid, easy and efficient test for serodiagnosis of VL.

## Author Summary

Visceral Leishmaniasis (VL) is a neglected tropical disease for which serodiagnostic tests are available, but not yet widely implemented in rural areas. The rK39 recombinant protein is derived from a kinesin-like protein of parasites belonging to the *Leishmania donovani* complex, and has been used in the last two decades for the serodiagnosis of VL. We present here a systematic review and meta-analysis of studies evaluating serologic assays (rK39 strip-test, rK39 ELISA, Direct Agglutination Test [DAT], Indirect Immunofluorescence test [IFAT] and ELISA with a promastigote antigen preparation [p-ELISA]) to diagnose VL to determine the accuracy of rK39 antigen in comparison to the use of other antigen preparations. Fourteen papers fulfilled the inclusion and exclusion selection criteria. The summarized sensitivity for the rK39-ELISA was 92% followed by IFAT 88% and p-ELISA 87%. The summarized specificity for the three diagnostic tests was 81%, 90%, and 77%. Studies comparing the rK39 strip test with DAT found a similar sensitivity (94%) and specificity (89%). However, the rK39 strip test was more specific than the IFAT and p-ELISA. In conclusion, we found the rK39 protein used either in a strip test or in an ELISA is a good choice for the serodiagnosis of VL.

## Introduction

Visceral Leishmaniasis (VL) is a neglected tropical disease for which a simple and quick diagnostic test is available, but not yet widely implemented in rural areas [1]. Although several different serodiagnostic test formats exist, many of them have not been validated in prospective field studies [2]. In addition, only a limited number of well conducted trials comparing the different types of tests have been published [2]. This lack of data has limited the routine use of these tests in many settings.

The optimal test for serologic diagnosis is one that is easy to use, cheap to make, and has both a high sensitivity and specificity. The use of crude antigens for the serodiagnosis of tropical diseases is often limited by the difficulty in producing large quantities of the antigen, and therefore can be difficult to make in a standardized manner. However, once a useful diagnostic antigen has been identified, modern molecular biology techniques allow the antigen to be manufactured as a recombinant protein in a standardized manner with an advantage over crude lysate undefined antigen used in DAT. In the recent past, numerous recombinant antigens have become available for use in serodiagnostic testing of leishmaniasis [3]. Molecular diagnostic assays for leishmaniasis using polymerase chain reaction (PCR) targeting multi-copy genes, (e.g., rRNA, kinetoplastDNA (kDNA) minicircles) have been developed. Sensitivity and specificity of these tests depend upon the region targeted, with a recent study finding a peripheral blood PCR assay having an overall sensitivity of 98.5% [4]. However, nucleic acid testing is currently difficult to perform in many clinical labs in the developing world.

The currently available serodiagnostic tests for VL have been based on four major formats: Direct agglutination (DAT), indirect immunofluorescence (IFAT), ELISA and immunocromatography [3], [5], [6]. The DAT and IFAT classically utilize whole promastigotes to screen for antibodies, while the p-ELISA uses a crude lysate of promastigotes. Immunochromatographic tests, and a newer ELISA have been developed using the recombinant protein rK39, which is a kinesin-like gene found in *Leishmania chagasi*. Recently, the WHO Special Program for Research and Training in Tropical Disease (TDR) evaluated five different immunochromatographic tests utilizing either rK39 or rKE16, a recombinant protein developed from the kinesin gene of a *Leishmania donovoni* isolate [7]. Testing was performed in East Africa, Brazil and on the Indian subcontinent, and sensitivities ranged from 36.8–100% and specificities from 90.8–100%. No test was the clear winner across all regions and conditions. In addition, since comparisons between different test types are limited, it remains unclear even which type of serologic test is most optimal for use in the diagnosis of VL.

Meta-analyses are excellent tools to evaluate multiple studies with similar goals but varying methods [8]. We performed a meta-analysis of studies evaluating serodiagnostic tests for VL to determine the accuracy of rK39 antigen based serodiagnostic tests in comparison to other available serodiagnostic tests for the diagnosis of VL.

## Methods

### Design of study

This is a systematic review of the literature using the Cochrane recommendations to compare the sensitivity and specificity of serodiagnostic tests using rK39 antigen in the ELISA and strip test formats against crude lysate *Leishmania* antigen used in an ELISA or whole promastigote parasite as antigen used in DAT and IFAT. Three hundred fifty-two papers have been published to date evaluating the rK39 antigen as diagnostic tool for serodiagnosis of VL. Within these reports, significant discrepancies in sensitivity and specificity are found, likely due to a multitude of reasons including: variability in testing methods, testing performed in different geographic regions, different sources for the tests, and lack of homogeneity of the studied population. Applying the selection criteria described below, we included 14 studies in this meta-analysis.

Inclusion criteria were: a) have a full description of accuracy of the diagnostic test, b) that were performed on human specimens, c) include a rK39 antigen based test, d) direct demonstration of *Leishmania* parasites as the confirmatory diagnostic method for VL.

**Figure 1:**
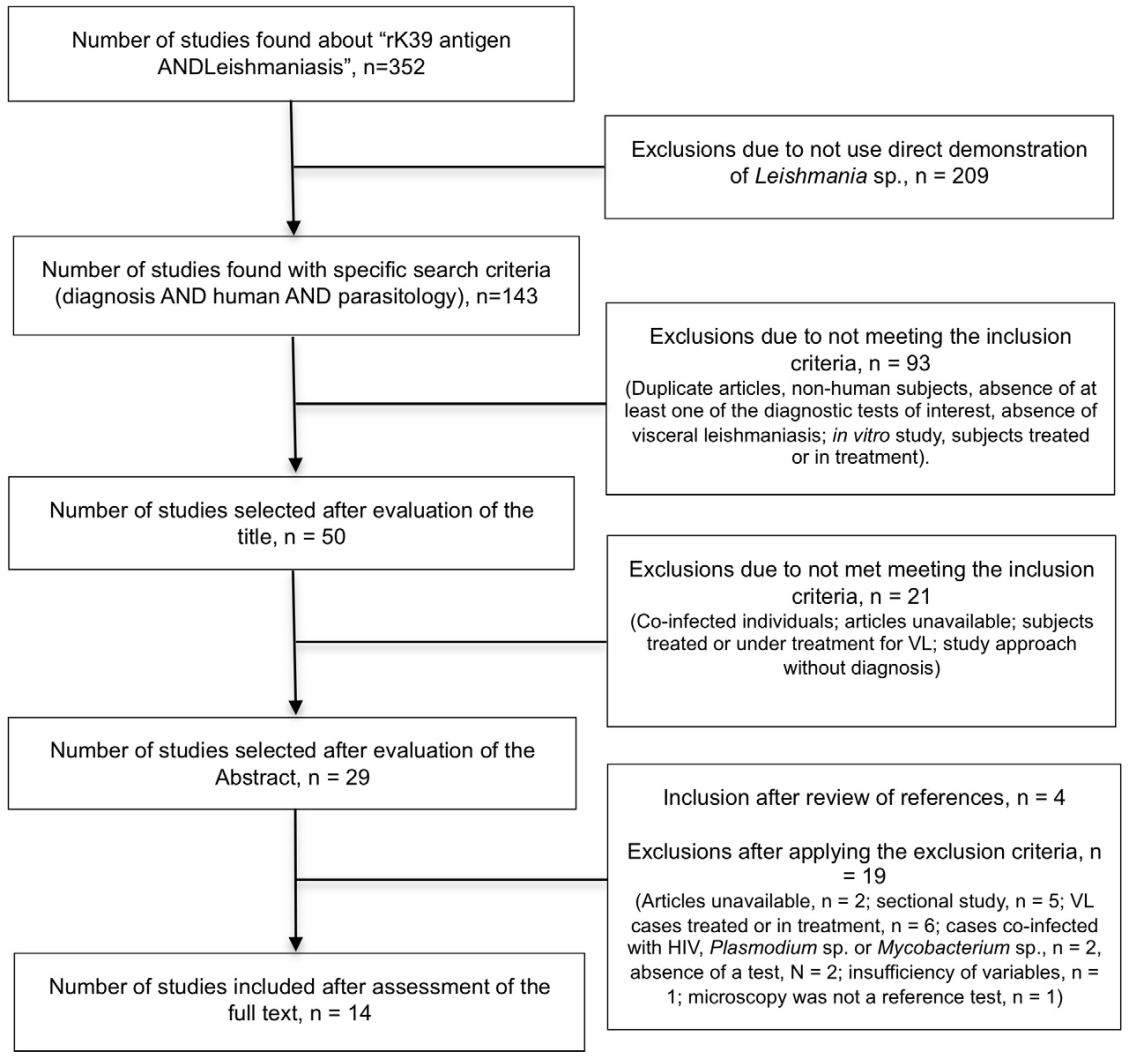
Algorithm for selection of studies.

Exclusion criteria were: a) insufficient primary and/or secondary data information of (lack information about sensitivity and specificity), b) lack of inclusion of a control group, c) inclusion of co-infections with HIV, d) inclusion of subjects that were receiving or had received VL treatment, e) full text article unavailable, f) written in a language other than English, Spanish and Portuguese. Figure 1 describes the process utilized for selection of studies for the meta-analysis.

### Source of data

Data were obtained by a search of the published literature cited in *Medline*, *Lilacs*, *Scopus*, *Isis Web of Science*, *Cochrane Library* accessed on June 1, 2011.

### Search strategy

Our search strategy utilized the following key words: rK39 antigen, Visceral Leishmaniasis, parasitology, accuracy test and human. We also utilized the suggested synonymous terms for our search. i) Initial screening of manuscripts was performed by selecting those with titles related to our subject of study ii) Each manuscript selected by title then had its abstract reviewed for inclusion or exclusion based upon the variables reported in the results. iii) Selected studies the had the full text reviewed to collect existing primary variables of true positive and false positive, true negative and false negative data presented in each study that allowed re-calculation of sensitivities and specificities, likelihood, predictive values and confidence intervals. Also, careful reviews of the references in each selected manuscript were performed to rescue those not selected by search strategy. This literature review was performed by two independent reviewers. If the reviewers were in consensus, the manuscript was accepted for the meta-analysis, if there was disagreement, a third reviewer was consulted. The QUADAS (*Quality in Diagnostic Accuracy Studies*) protocol was used to evaluate the quality of each study [9].

### Statistic analysis

Receiver operating characteristic (ROC) plot were constructed using the R Statistical Software package.

## Results

Our initial electronic search identified 352 articles that were related to the serodiagnosis of leishmaniasis. Filtering these articles to those of interest to us, focusing on human studies of rK39, the list was narrowed to143 studies. Among these, only 50 evaluated the sensitivity and/or specificity of serodiagnostic testing for VL. Finally, 40 of these studies were excluded due to duplicity (same study found in different sources), inclusion of subjects with HIV/*Leishmania* co-infection, study of cutaneous leishmaniasis, lack of evaluation of at least one of the diagnostic tests of interest, and evaluation of subjects that were already treated or in treatment. We included an additional four articles after a review of references from our selected articles.

### Description of selected studies

A total of 5,548 individuals were included into the meta-analysis from the 14 included studies: 2,097 cases of VL and 3,451 controls. The distribution of studies according to region was as follows: Brazil (n = 5); Indian (n = 4); Nepal (n = 2); Tunis, Italy, and Kuwait (n = 1 each). The manuscripts were published during the period between 1998 to 2011. Thirteen studies reported results of strip tests: Sundar 1998 [10], Sundar 2007 [11], Saghrouni 2009 [12], Romero 2009 [13], Mathur 2005 [14], Mandal 2008 [15], Iqbal 2002 [16], de Assis 2008 [17], de Assis 2011 [18], Chappuis 2003 [19], Carvalho 2003 [20], Brandonisio 2002 [21], and Boelaert 2004 [22]. Three reported results with the rk39-ELISA: de Assis 2008 [17], Pedras 2008 [23] and Romero 2009 [13]. IFAT results were reported from seven studies Boelaert 2004 [22], Brandonisio 2002 [21], de Assis 2008 [17], Iqbal 2002 [16], Pedras 2008 [23], Romero 2009 [13] and Saghrouni 2009 [12]; ELISA with crude promastigote lysate results were reported from six studies: Carvalho 2003 [20], de Assis 2008 [17], Mathur 2005 [14], Pedras 2008 [23], Romero 2009 [13], and Mandal 2008 [15]; and finally DAT results were reported from six studies: Boelaert 2004 [22], Chappuis 2003 [19], Pedras 2008 [23], Sundar 2007 [11], de Assis 2011 [18], and Mandal 2008 [15].

A total of 12 studies fulfilled equal or greater than 10 questions from the 14 QUADAS questions. The studies of Brandonisio 2002 [21] and Mandal 2008 [15] fulfilled 8 and 7 questions respectively.

### Measurement of accuracy

The summarized sensitivity of the 13 studies evaluating the rK39 antigen strip test was 92% [91.49–92.92] and the summarized specificity was 95% [94.30–95.48]. The likelihood ratio of a positive test (LR+) was found to be 18.042 [18.03–18.06] and the likelihood ratio of a negative test (LR−) was found to be 0.082 [−0.13–0.29].

These 13 studies were stratified according to which technique were compared. i) Five studies compared the strip test vs p-ELISA: Carvalho 2003 [20], de Assis 2008 [17], Mathur 2005 [14], Romero 2009 [13] and Mandal 2008 [15]; ii) five compared the strip test vs DAT: Boelaert 2004 [22], Chappuis 2003 [19], Sundar 2007 [11], de Assis 2011 [18], Mandal 2008 [15]; and iii) six compared the strip test vs IFAT: Boelaert 2004 [22], Brandonisio 2002 [21], de Assis 2008 [17], Iqbal 2002 [16], Romero 2009 [13] and Saghrouni 2009 [12].

**Figure 2:**
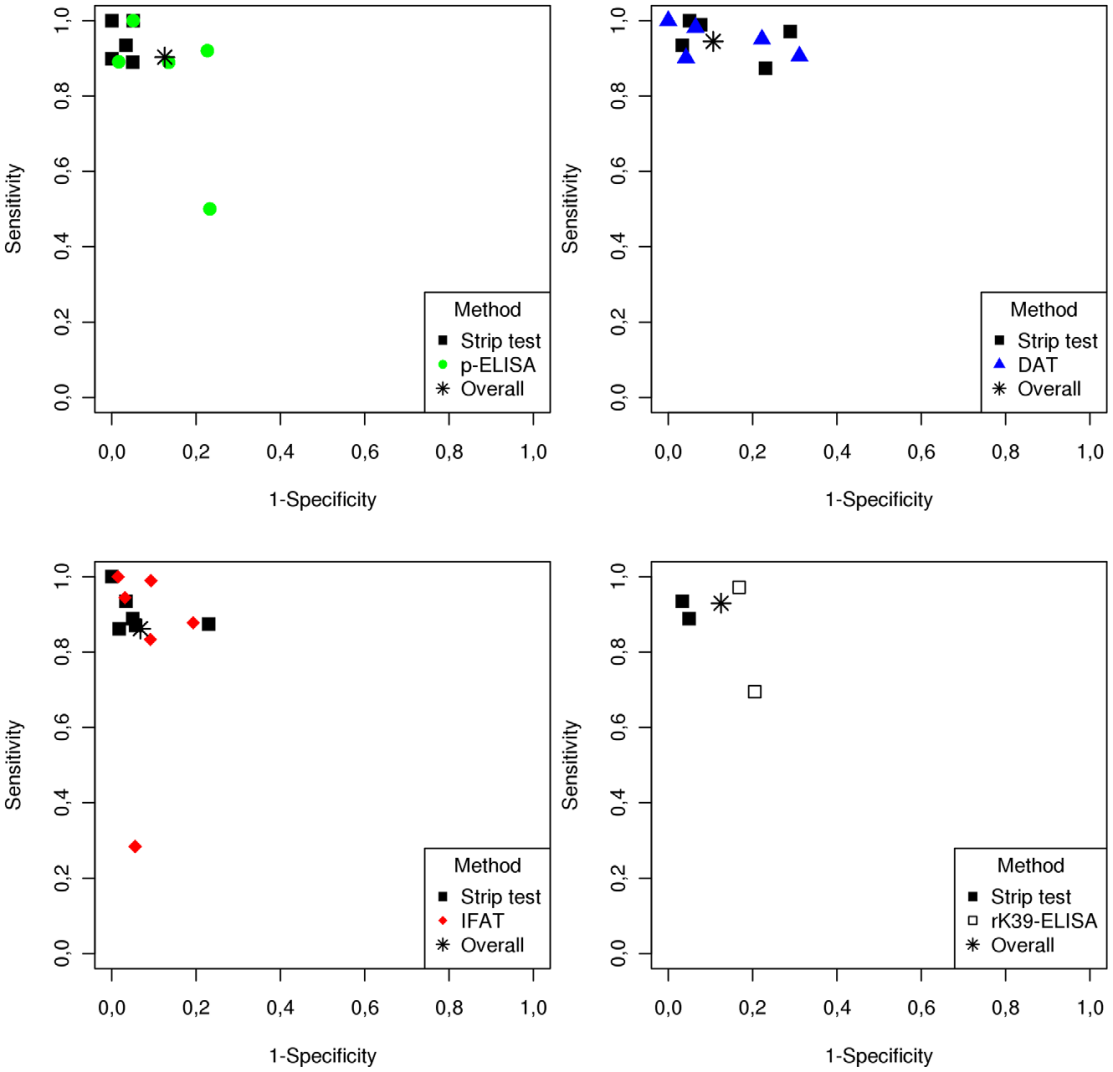
ROC plot comparing the performance of the rK39 strip test to serologic techniques utilizing promastigote antigens for diagnosis of Visceral Leishmaniasis.

Figure 2 demonstrates the ROC plot for comparison of sensitivity and specificity of the strip test and other test formats. The ROC plot for the strip test vs p-ELISA demonstrating a sensitivity, specificity and a positive likelihood ratio (LR+) of 93%, 96%, and 21.16 for the strip test and 88%, 79%, and 4.14 for p-ELISA, respectively. The study by Romero et al had sensitivity and specificity values that were quite different from the other studies (sensitivity of 50% and specificity of 77%). The exclusion of the Romero study for ROC analysis increased the specificity of strip test 99% and (LR+) to 68.77, but did not alter the sensitivity, and increased the sensitivity to 91%, specificity 88%, (LR+) to 7.81 for the p-ELISA. ROC plots for the strip test vs. DAT demonstrated that the sensitivities and specificities were very close for both tests (94.23%, 89.97% and LR+ 9.39 for DAT and 94.48% and 88.75% and LR+ 8.40 for the strip test). The ROC plot comparison of the strip test vs. IFAT revealed a better performance of strip test than IFAT with a sensitivity, specificity and LR+ of 87%, 95% and 16.89 for the strip test vs 84%, 92% and 10.01 for the IFAT. Only two studies compared the strip test vs. the rK39-ELISA: de Assis 2011 [18] and Romero 2009 [13]. The summarized sensitivity of the tests were similar (93% for both) and the specificity of each test was 95% and 80% respectively. This resulted in a higher LR+ for the strip test than the rK39-ELISA (19.24 vs. 4.61 respectively).

Only three studies evaluated the rK39 antigen used in the ELISA format: de Assis 2011 [18], Pedras 2008 [23] and Romero 2009 [13]. The pooled characteristics of the studies were: sensitivity of 92%, specificity of 80% and LR+ of 4.73.

**Figure 3:**
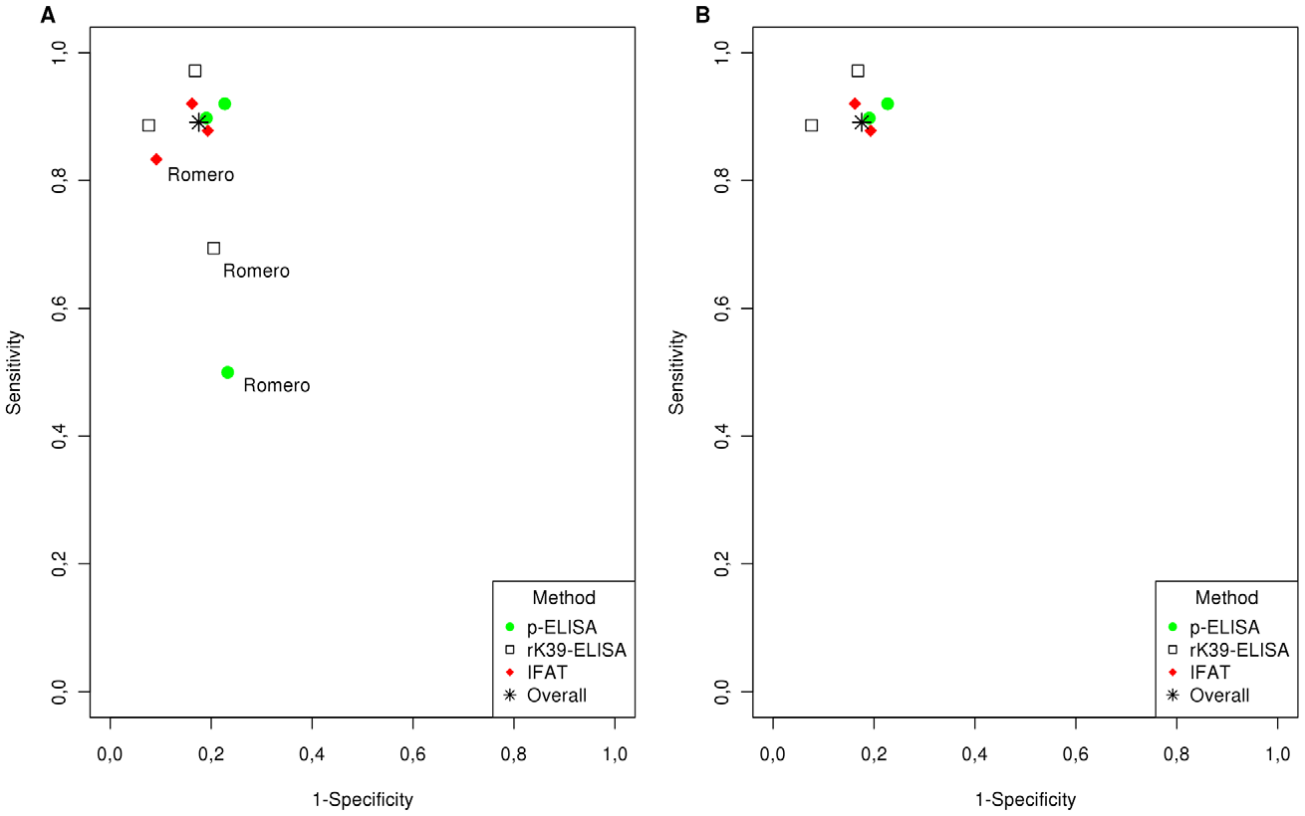
ROC plot comparing the performance of the rK39-ELISA to the p-ELISA and IFAT for the diagnosis of Visceral Leishmaniasis. (**A**) Analysis including all studies that met our selection criteria. (**B**) Analysis after removal of the outlier study.

ROC plot comparison of rK39-ELISA, p-ELISA and IFAT is presented in figure 3. Summarized sensitivity results for the three tests are 92%, 87% and 88% and summarized specificity results are 81%, 77% and 90% respectively. However, evaluation of the Romero study [13] noted discrepancies between the raw data and in the sensitivity and confidence intervals (Table 1). The ROC plot constructed excluding Romero's study is presented in figure 2B.

**Table 1:** Comparison of the sensitivity, specificity and likelihood ratio of tests.

Comparison	n	Test	Summary measures			
			S%[CI]	Sp%[CI]	LR+[CI]	LR−[CI]
Strip test vs p-ELISA	5	Strip test	92.89 [91.85–93.93]	95.61 [94.78–96.44]	21.16 [21.14–21-19]	0.07 [−0.32–0.47]
		p-ELISA	87.62 [86.28–88.95]	78.86 [77.21–80.52]	4.14 [4.10–4.19]	0.16 [−0.11–0.43]
	4*	Strip test	93.25 [91.53–94.97]	98.64 [97.85–99.44]	68.77 [68.74–68.80]	0.07 [−0.64–0.78]
		p-ELISA	91.14 [89.20–93.09]	88.32 [86.13–90.52]	7.81 [7.76–7.86]	0.10 [−0.34–0.54]
Strip test vs DAT	5	Strip test	94.48 [93.29–95.67]	88.75 [87.11–90.40]	8.40 [8.37–8.43]	0.06 [−1.03–1.15]
		DAT	94.23 [93.01–95.45]	89.97 [88.40–91.53]	9.39 [9.36–9.42]	0.06 [−0.30–0.43]
Strip test vs IFAT	6	Strip test	88.60 [87.56–89.65]	94.75 [94.02–95.49]	16.89 [16.87–16.91]	0.12 [−0.12–0.36]
		IFAT	83.76 [82.55–84.97]	91.63 [90.72–92.54]	10.01 [9.98–10.04]	0.18 [−0.01–0.37]
rK39-ELISA vs p-ELISA vs IFAT	3	rK39-ELISA	91.99 [90.81–93.17]	80.55 [78.83–82.27]	4.73 [4.69–4.77]	0.10 [−0.28–0.47]
		p-ELISA	86.94 [85.48–88.41]	77.03 [75.20–78.85]	3.78 [3.74–3.83]	0.17 [−0.12–0.46]
		IFAT	88.43 [87.04–89.82]	89.72 [88.40–91.04]	8.60 [8.56–8.64]	0.13 [−0.20–0.46]
	2*	rK39-ELISA	94.68 [92.77–96.60]	87.50 [84.67–90.33]	7.57 [7.52–7.63]	0.06 [−0.53–0.65]
		p-ELISA	91.36 [88.96–93.77]	79.02 [75.53–82.50]	4.3 [4.28–4.43]	0.11 [−0.34–0.56]
		IFAT	89.04 [86.36–91.71]	82.14 [78.87–85.42]	4.99 [4.91–5.06]	0.13 [−0.29–0.56]
rK39-ELISA vs Strip test	2	rK39-ELISA	93.17 [92.02–94.32]	79.77 [77.94–81.61]	4.61 [4.56–4.65]	0.09 [−0.38–0.55]
		Strip test	92.77 [91.59–93.95]	95.18 [94.20–96.16]	19.24 [19.20–19.28]	0.08 [0.04–0.57]

p-ELISA = enzyme immunoassay with antigens of promastigotes of *Leishmania* sp. DAT = direct agglutination test, IFAT = indirect immunofluorescence; rK39-ELISA = enzyme immunoassay with rK39 antigen, n = number of studies included in the analysis, S = sensitivity, Sp = specificity; LR+ = positive likelihood ratio, LR− = negative likelihood ratio. CI = confidence interval.

*Analysis in which the study by Romero et al. was excluded because of discrepancies noted in the published data.

### Analysis of heterogeneity of the study

**Figure 4:**
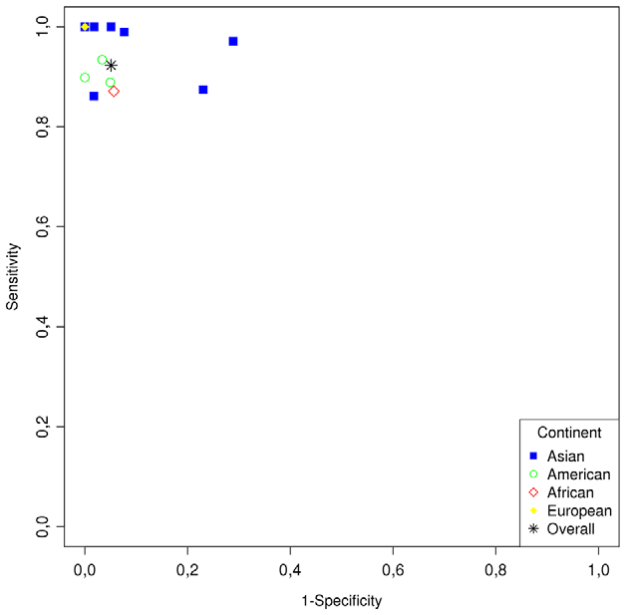
ROC plot showing the heterogeneity of strip test by continent.

From the thirteen studies that utilized the strip test format, nine used the Kalazar Detect™ Test for VL manufactured by Inbios (Seattle, USA): Sundar 2007 [11], Saghrouni 2009 [12], Romero 2009 [13], Mathur 2005 [14], Mandal 2008 [15], Chappuis 2003 [19], Carvalho 2003 [20], Brandonisio 2002 [21], and Boelaert 2004 [22]. Two studies used DiaMed-IT's Leish: de Assis 2008 [17], de Assis 2011 [18]; one study used the Leishmania rapid test strip-test from Intersep: Iqbal 2002 [16]; and one study the recombinant K39 strip test from Arista Biologicals: Sundar 1998 [10]. The best performances in a single study for a strip test were demonstrated with the recombinant K39 strip test from Arista Biologicals, followed by the strip test from Inbios. However, there was considerable variability in the results from the studies conducted with the Inbios strip test. Some of the variability in sensitivity and specificity has been shown to be associated with the geographic region where the test is being performed [7]. The best receiver operating characteristic (ROC) plot for the Inbios strip test was demonstrated by the results of Brandonisio 2002 [21] who evaluated the test in Italy (Figure 4).

### Comparison by source of *Leishmania* antigens and tests details

Three studies evaluated a p-ELISA using *L. chagasi* promastigotes as the source of antigen: Carvalho 2003 [20] de Assis 2008 [17], and Pedras, 2008 [23]; two studies used *L. infantum*: Mathur 2005 [14], Mandal 2008 [15]: and one study *L. amazonensis*: Romero 2009 [13]. The ROC plot was better for the studies that used *L. infantum* with the relative order of *L. infantum*>*L. chagasi>L. amazonensis*.

Cut off dilution for a positive test on DAT varies from 1∶800 to 1∶6400. The ROC plot demonstrated that the order of dilutions was 1∶800>1∶1600>1∶3200>1∶6400. For IFAT the cut off dilution varies from 1∶32 to 1∶400, ROC plot demonstrated that the order of dilutions was 1∶400>1∶160>1∶80>1∶40>1∶32.

## Discussion

This meta-analysis utilized rigorous selection criteria to select published papers that compared four different serodiagnositic tests currently being utilized in endemic areas for serodiagnosis of VL. We found that recombinant K39 protein used either in a strip test or ELISA format and the DAT using whole promastigote antigen are the most accurate tests for serodiagnosis of VL.

During the past 70 years where various strategies have been used for the serodiagnosis of VL, there have been waves of enthusiasm favoring one technique over others [24], [25]. The formol-gel test (FGT) based on gelling and opacification of the serum from a patient with VL in the presence of formaldehyde was the only bedside test available in earlier days of last century to confirm a diagnosis of VL in patients suspected to have Kala-azar [22]. Early on it was discovered that high levels of specific antibodies against *L. donovani* spp. and a polyclonal B cell activation classified VL as one of the few diseases resembling multiple myeloma because of the high levels of globulins found in patients' serum [26]. This property of VL allowed for the development of serodiagnostic tests such as the DAT. The DAT easily can detect high titers up to or higher 1/64.000 dilution folds in a serum of most VL patients [27], and there are many comparative studies that have demonstrated that DAT is a good option to help physicians in endemic areas to confirm the diagnosis of VL in patients with suggestive symptoms, and when examination for *Leishmania* parasites in a bone marrow or spleen aspirate biopsies is not available [5], [6], [28], [29], [30]. Why has the DAT not been developed into an affordable commercial test available for use in endemic areas? The WHO attempted to push the development of such a test in the early 1990s, but was not successful due to the following reasons: 1) an essential part of the test is the growth of parasites, and the variability in the techniques and preservation of the antigen can lead to variation in test results and 2) high levels of cross reactivity with other trypanosomatides exist [6], [31], [32], [33], [34], [35]. The IFAT for VL is a very sensitive test, but far from being a diagnostic tool in endemic areas, as it has similar limitations in terms of antigen preparation, and in addition requires the use of special microscope equipment [36]. On the other hand, ELISA has been extensively used for serological diagnosis in VL. However, the sensitivity and specificity of this test is variable depending upon the antigen chosen for use [29], [35], [37]. More recently, third generation tests for the serodiagnosis of VL have utilized two basic formats: ELISA and immunochromatography [2], [36]. A 2003 comparison of 11 defined recombinant or synthetic proteins with soluble *Leishmania* antigen (SLA) in an ELISA format revealed that the best antigen was rK39 with 100% sensitivity and 97% specificity in the diagnosis of VL [38]. The recombinant protein K39 (rK39) is a repeated 39-amino acid sequence derived from a gene cloned from *L. chagasi* and expressed in *E. coli*. The protein is related to the kinesin family of proteins, has a high epitope density, and is present in high amount in the amastigotes forms of *L. donovani* complex [39]. rK39 was first demonstrated to be an indicator of disease in *L. chagasi* infected patients [40]. More recently, the rK39 antigen has been developed into a strip test for the serodiagnosis of VL [10], [19], [41], [42], [43].

When comparing the rK39 strip test and the DAT, several issues arise. The DAT has the ability to detect low levels of antibodies due to the mosaic of antigens present in the extract. This sensitivity can come at a cost in specificity, as some of these antigens are cross reactive, and therefore careful attention needs to be placed on determining the cut-off values for a positive test [44]. Strip tests also have limitations, as with other serologic tests, patients can have antibody present for months after cure of disease, and also the tests can detect antibodies in the sera of asymptomatic patients [24]. False negative results have been reported and may vary from location to location [36]. In this review, 154 out of 1884 stored samples from clinically ill patients with positive amastigotes in bone marrow aspirate did not react with the strip test. This finding could potentially be explained by variations in sample storage protocols [45].

A previous meta-analysis comparing the DAT and strip test including 30 papers evaluating the DAT 13 evaluating the rK39 strip found that tests are comparable. The DAT was found to be 1% most sensitive and 2% more specific than strip test, but this analysis did not report ROC comparison of sensitivity and specificity because not all studies selected performed both techniques [46]. One important criteria of our meta-analysis study was the inclusion of only studies that compared both methods in each individual study. In addition our meta-analysis included comparison of ELISA and IFAT. In our study the best performance of the strip test were from those studies carried out in Europe, different from Chappuis's meta-analysis which found that sensitivity seemed higher and more homogenous in studies carried out in South Asia [46].

A newly developed assay based on the detection of antibodies to the rk28 fusion protein reported a very promising sensitivity and specificity (96% and 98% respectively) of ELISA to detect anti-*Leishmania* antibody in sera from VL patients. However, both the rK39 and rK28 antigens demonstrated similar areas under the ROC curves [47], [48].

Meta-analysis is an important tool that drives direction for best evidences in medicine by comparing studies done in different places, environments and populations as long as the same question was used by different investigators. But, unfortunately when we focus the meta-analysis on sensitivities and specificities of serological tests the heterogeneity introduced by variations in diagnostic thresholds becomes an important limitation of this analytic tool [49]. We suggest that investigators in endemic areas should consider using both rK39 strip test and DAT prior to initiating anti-*Leishmania* treatment when demonstration of the parasite in bone marrow or spleen aspirate biopsies is not available.
